# Optimization of Phlorotannins Extraction from *Fucus vesiculosus* and Evaluation of Their Potential to Prevent Metabolic Disorders

**DOI:** 10.3390/md17030162

**Published:** 2019-03-08

**Authors:** Marcelo D. Catarino, Artur M. S. Silva, Nuno Mateus, Susana M. Cardoso

**Affiliations:** 1 QOPNA & LAQV-REQUIMTE, Department of Chemistry, University of Aveiro, 3810-193 Aveiro, Portugal; mcatarino@ua.pt (M.D.C.); artur.silva@ua.pt (A.M.S.S.); 2 REQUIMTE/LAQV, Department of Chemistry and Biochemistry, Faculty of Sciences, University of Porto, 4169-007 Porto, Portugal; nbmateus@fc.up.pt

**Keywords:** *Fucus vesiculosus*, phlorotannins, α-glucosidase, α-amylase, pancreatic lipase, diabetes, obesity, response surface methodology

## Abstract

Phlorotannins are phloroglucinol-based phenolic compounds, occurring particularly in brown macroalgae, that have been recognized for their promising bioactive properties. In this study, the extraction of phlorotannins from *Fucus vesiculosus* was evaluated with particular emphasis on the influential parameters, including the solvent concentration, solvent-solid ratio, extraction temperature and extraction time, using a single-factor design followed by a Box-Behnken design. The maximum total phlorotannin content, determined using the 2,4-dimethoxybenzaldehyde (DMBA) method, corresponded to 2.92 ± 0.05 mg of phloroglucinol equivalents/g dry seaweed (mg PGE/g DS), and was achieved for extracts carried out with acetone 67% (v/v), a solvent-solid ratio of 70 mL/g and temperature at 25 °C. This crude extract, together with a semi-purified phlorotannin fraction, were further evaluated for their anti-enzymatic capacity against α-glucosidase, α-amylase and pancreatic lipase, both showing promising inhibitory effects, particularly against α-glucosidase for which a greater inhibitory effect was observed compared to the pharmaceutical drug acarbose (IC_50_ = 4.5 ± 0.8 and 0.82 ± 0.3 μg/mL, respectively, against 206.6 ± 25.1 μg/mL). Additionally, the ultra-high-pressure liquid chromatography coupled to mass spectrometry (UHPLC-MS) analysis carried out on the ethyl acetate fraction revealed the presence of fucols, fucophlorethols, fuhalols and several other phlorotannin derivatives. Moreover, possible new phlorotannin compounds, including fucofurodiphlorethol, fucofurotriphlorethol and fucofuropentaphlorethol, have been tentatively identified in this extract. Overall, this study provides evidence that *F. vesiculosus* phlorotannin-rich extracts hold potential for the management of the activity of α-glucosidase, α-amylase and pancreatic lipase, which are well known to be linked to metabolic disorders such as diabetes and obesity.

## 1. Introduction

Phlorotannins are phenolic compounds consisting of dehydro-oligomers or dehydro-polymers formed through the C–C and/or C–O–C oxidative coupling of phloroglucinol (1,3,5-trihydroxybenzene) monomeric units that is known to occur particularly on brown macroalgae [1]. Indeed, species such *Eisenia bicyclis*, *Ishige okamurae*, *Sargassum thunbergii*, *Sargassum fusiforme*, *Undaria pinnatifida* and *Ascophyllum nodosum*, as well as algae belonging to the genera *Cystophora, Ecklonia* and *Fucus*, are examples of marine brown algae that have been reported as good sources of phlorotannins with promising health benefits including antioxidant, anti-inflammatory, antibacterial, anticancer, antidiabetic and anti-obesity properties [2]. For this reason, during recent years, many researchers have raised their interest in algae phenolics, triggering an exponential increase in the number of phlorotannins-related publications.

Among these algae, *Fucus vesiculosus,* commonly known as bladderwrack, appears as an interesting source of such compounds [3]. This species is widely distributed in the intertidal areas of many cold and warm temperate regions in the Northern Hemisphere and consists of a holdfast, a small stipe, and flattened dichotomously-branched blades with several air bladders that keep them afloat in a vertical position when submerged [4]. *F. vesiculosus* has long been harvested and used as a food source not only in far East Asian countries, but also in some coastal countries in Western Europe and in Alaska [5], due to its remarkable therapeutic properties mainly for treating goiter and obesity [6], and also cellulite, blood clot formations, rheumatoid arthritis, asthma, atherosclerosis, diabetes, psoriasis and skin diseases, cancer and other oxidative and inflammatory related conditions [7,8,9,10]. According to what Holdt and Kraan revised [11], the genus *Fucus* are among the brown macroalgae that accumulate the highest amounts of phlorotannins, reaching up to 12% dry weight, although their concentrations are highly susceptible to distinct factors including seasonality, solar exposure, salinity or geographical origin. Phlorotannin extracts of this genus have been described for their promising antioxidant, anti-inflammatory and antitumor activities [12], and there are even some studies that reported the potential of using *F. vesiculosus* extracts as functional ingredients for the development of novel and improved foods, mainly in the field of dairy and seafood products [13,14,15]. Interestingly, despite several authors have reported promising antidiabetic effects for phlorotannins, only a few studies have approached the antidiabetic and anti-obesity potential of phlorotannin extracts from *F. vesiculosus*. In this subject, the inhibitory capacity against α-glucosidase is perhaps the most well studied effect. Indeed, both ethanol 96% and acetone 70% extracts from *F. vesiculosus* collected in France were found to be the strongest inhibitors of this enzyme amongst another six brown algae species tested [16]. Likewise, Lordan et al. [17] reported good inhibitory effects for 80% ethanol extracts from Irish *F. vesiculosus* against both α-glucosidase and α-amylase. Moreover, recent studies from our group showed that different crude extracts from the *F. vesiculosus* of the northern Portuguese coast, particularly that of acetone 70%, had promising inhibitory effects against α-glucosidase [18]. In turn, only the study of Chater et al. [19] has previously suggested that ethanolic and aqueous extracts from *F. vesiculosus* collected on the west coast of Scotland could exert some potential inhibitory effects towards pancreatic lipase, although the lack of comparison with a specific inhibitor does not allow the drawing of relevant conclusions. 

Due to their complex chemical structure, susceptibility to oxidation and interaction with other matrix components, the extraction of phlorotannins can be a demanding task. Many factors, such as solvent composition, solvent polarity, time of extraction, temperature, solvent-solid ratio and particle size, may significantly influence the solid–liquid extraction of phenolic compounds [20]. The most common protocols used for the extraction of phlorotannins are based on aqueous mixtures of acetone, ethanol or methanol [21,22,23]. Indeed, Koivikko et al. [24] studied the influence of different solvents namely ethyl acetate, ethanol, methanol, acetone and water on the amount of phlorotannins extracted from *F. vesiculosus* and found that a water/acetone (30:70) mixture was the most suitable solvent system for the extraction of phlorotannins from this species. However, according to our knowledge, a proper study to evaluate the effects of cross interactions between different factors on *F. vesiculosus* phlorotannins extraction has never been performed before.

In this context, this study intended to clarify the effects of four different extraction parameters, namely solvent concentration, solvent-solid ratio, temperature and time, on the total phlorotannin content of *F. vesiculosus* collected on the northern Portuguese coast and to optimize the recovery yields of these compounds using a Box-Behnken design (BBD), i.e., one instrument of response surface methodology (RSM) that uses quantitative data from an appropriate experimental design to determine or simultaneously solve a multivariate equation. Additionally, the optimized crude extract, as well as a subsequent purified phlorotannin fraction, were further evaluated with regard to their antidiabetic and anti-obesity potential through the inhibition of α-glucosidase, α-amylase and pancreatic lipase. Finally, a comprehensive interpretation of the UHPLC-DAD-ESI-MS^n^ data will allow new insights into the composition of phlorotannins from the genus *Fucus*.

## 2. Results

### 2.1. Single-Factor Experiments

Prior to implementing the BBD, preliminary single-factor experiments concerning the relevant variables that could affect the phlorotannin extraction were conducted to narrow the range of selected factors in the BBD experiment.

#### 2.1.1. Effect of the Acetone Concentration on Total Phlorotannin Content (TPhC)

**Figure 1:**
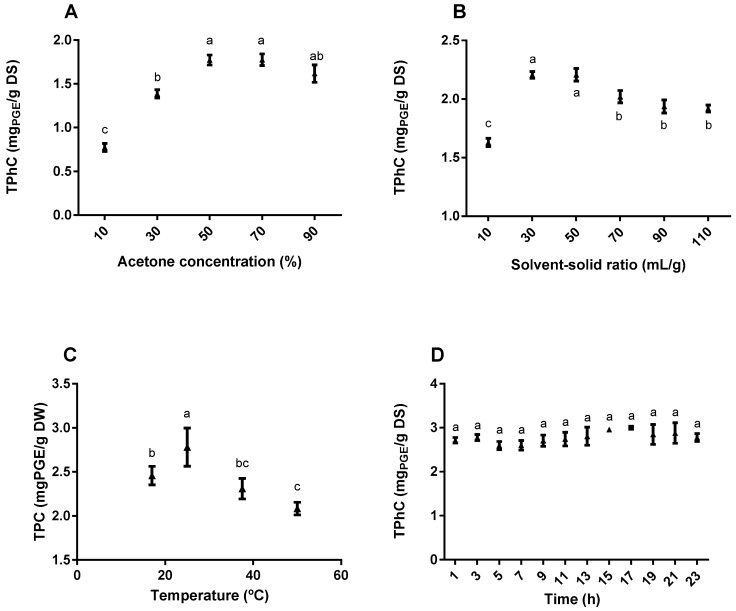
Effect of (**A**) acetone concentration, (**B**) solvent-solid ratio, (**C**) temperature and (**D**) extracting time on the total phlorotannin content of *F. vesiculosus* extracts in the single-factor experiments. Initial extraction conditions consisted of 70% acetone, in a proportion of 1:20 (m:v) at room temperature during 24 h. Before moving to the next experiment, the previous condition was fixed at the point that showed the best total phlorotannin content (TPhC). Data represent the mean ± SEM of at least 3 independent assays and the results are expressed in mg of phloroglucinol equivalents/g of dried seaweed. Different letters represent statistical significance (one-way ANOVA followed by Tukey’s post hoc test; *p* ≤ 0.05).

Usually, phenolic compounds are easily soluble in solvents less polar than water, and therefore, the most common extractants used for these compounds are methanol, ethanol and acetone or aqueous mixtures of these. Particularly concerning phlorotannins, several studies have shown that the highest extraction yields were achieved with acetone [24,25,26,27,28]. These results were also in line with our preliminary experiments, which confirmed a superior recovery of TPhC with acetone compared with ethanol or methanol (data not shown). Therefore, different concentrations of this solvent were further tested in a range of 10 to 90% (v/v). As depicted in Figure 1A, the recovery of TPhC from *F. vesiculosus* increased proportionally for acetone concentrations between 10 to 50% (0.77 ± 0.05 to 1.77 ± 0.06 mg PGE/g DS) while maximum values were obtained for acetone concentrations between 50 to 70% (1.77 ± 0.06 and 1.73 ± 0.07 mg PGE/g DS, respectively). The increment of acetone above 70% caused a downward tendency in the TPhC of the extracts, suggesting that, in such conditions, the polarity of the solvent is above the ideal for phlorotannin extraction. Other authors demonstrated that concentrations between 40 to 80% of acetone were more effective than pure acetone or water to produce brown algae extracts with high content in phlorotannins [24,29,30,31]. Interestingly, Leyton et al. demonstrated that water was the best solvent for phlorotannin extraction in *Macrocystis pyrifera* [32]. However, for higher water percentages, polysaccharides and proteins are solubilized easily, resulting in more complex and/or less pure extracts that consequently require additional purification steps. In the particular case of *F. vesiculosus*, after comparing water, acetone and an aqueous mixture of 70% acetone, Koivikko et al. [24] concluded that the latter was the most effective solvent for phlorotannin extraction, which was further confirmed by Wang et al. [33], who showed that 70% acetone produced the extracts with the highest phlorotannin content and simultaneously the strongest antioxidant effects. Consequently, for the BBD experiment, an acetone concentration range between 30–70% was selected.

#### 2.1.2. Effect of the Solvent-Solid Ratio on TPhC 

Based on the mass transfer principle, the higher the volume of solvent used, the greater the concentration gradient will be, driving the transference of the solutes from the sample matrix to the external solvent [34]. In this study, the effect of different solvent-solid ratios ranging from 10 to 110 mL/g on the phlorotannin recovery from *F. vesiculosus* were examined (Figure 1B). Increasing phlorotannin recoveries were observed when the ratios varied from 10 to 30 mL/g (1.63 ± 0.03 to 2.21 ± 0.03 mg PGE/g DS). In turn, TPhC remained constant for ratios between 30 to 50 mL/g and and decreased for higher ratios. Similar findings have been previously reported by Boi et al. [35] who showed that the recovery of TPhC from *Sargassum serratum* gradually increased until the ratio reached 40 mL/g, after which it tended to decrease. Our results suggest that increasing volumes of solvent enhances the extraction of phlorotannins until reaching an equilibrium (between 30 to 50 mL/g) after which other compounds start to be co-extracted. Considering the results obtained for this experiment, the solvent-solid ratio interval selected for the BBD experiment was set at 30 to 70 mL/g.

#### 2.1.3. Effect of the Temperature on TPhC

Higher extraction temperatures are often used to improve extraction yields since they increase molecular movement and decrease solvents’ viscosity, making them more prone to penetrate the sample matrix and dissolve target compounds easily. On the other hand, for thermolabile compounds such as phenolics, the use of temperature may trigger their degradation and consequently hinder their extraction [36]. Therefore, it is necessary to select a proper extraction temperature that ensures the maximum extraction yields without damaging the target compounds. For this reason, five different extractions were conducted at room temperature (approximately 17 °C), 25, 37.5 and 50 °C. As depicted in Figure 1C, there is a significant increase in phlorotannin recovery when the extracting temperatures rise from room temperature to 25 °C (2.46 ± 0.05 to 2.78 ± 0.11 mg PGE/g DS), but higher temperatures caused a gradual decrease in TPhC from 2.78 ± 0.11 mg PGE/g DS (at 25 °C) to 2.03 ± 0.06 mg PGE/g DS (at 50 °C). Some authors have reported optimum temperatures between 50 to 60 °C for the extraction of phlorotannins from different brown seaweed species, including *Macrocystis pyrifera* [32] and *Saccharina japonica* [37]. However, it is possible that the phlorotannins present in the species herein studied, i.e., *F. vesiculosus*, are more thermolabile, which could explain why lower temperatures are more appropriate for their extraction. In fact, the curve observed in Figure 1C is identical to that reported by Li et al. [38], who also observed a significant enhancement in phlorotannin extraction from *S. fusiforme* when the temperature was increased from 15 to 25 °C followed by a drastic decline for temperatures above. Based on these results, the temperature interval chosen for testing in the BBD experiment was 15 to 35 °C. 

#### 2.1.4. Effect of Time on TPhC

The determination of an adequate extraction time is important not only for ensuring an efficient extraction of the target compounds, but also for minimizing energy and other associated costs [39]. Therefore, in this study the extraction time was monitored in periods of 2 h over 23 h to evaluate its effect on the phlorotannin content and antioxidant activity of the extracts. Figure 1D shows that the variation of the extraction time did not significantly influence the TPhC of the extracts, which remained constant at approximately 2.8 mg PGE/g DS from 1 to 23 h. It is possible that equilibrium for phlorotannin extraction is achieved for a time point below 60 min, which explains the inexistence of variations for the range of time periods tested. In a previous study carried out with *F. vesiculosus*, it was demonstrated that 1 h was enough to extract approximately 80% of the phlorotannins from this seaweed [24]. More recently, Kadam et al. [40] reported that the extraction of phenolic compounds from *A. nodosum*, another brown algae species belonging to Fucaceae, was not affected at all by time variations between 5 to 25 min. It is common to find in literature numerous studies where the extraction procedures used for phlorotannin recovery and analysis take about 24 h, which is very time consuming [41,42,43]. The results herein presented show that it is possible to reduce the extraction time of *F. vesiculosus* for 3 h or less without compromising the total phlorotannin content of the extracts, and it is for this reason that the extraction time used for the BBD experiment was fixed at 3 h.

### 2.2. Analysis of the Response Surface Methodology

#### 2.2.1. Fitting the Model

Fitting the models for TPhC values is important to assess how precisely this response surface method can predict the ideal variances and determine the correlations of the selected parameters to the corresponding response. The experimental values obtained for the extracts’ TPhC (see Section 3.2.2.) were fitted to a quadratic polynomial model (Equation (1)) and used to study the correlations between the independent variables and corresponding responses, as well as to determine the optimum conditions for maximization of phlorotannin extraction from *F. vesiculosus*. Through an analysis of variance (ANOVA), it was possible to determine the significance of the coefficients, which can be observed in Table 1. The results show that the independent variables with a higher impact on TPhC were the acetone concentration (*p* < 0.001) followed by the solvent-solid ratio (*p* < 0.01), whereas no effect on temperature was seen. The acetone concentration also demonstrated a significant quadratic effect on phlorotannin recoveries (*p* < 0.001) as well as on the interaction between the acetone concentration and solvent-solid ratio (*p* < 0.001).

TPhC = 2.69 + 0.31X_1_ + 0.08X_2_ + 0.02X_3_ − 0.25X_1_^2^ + 0.02X_2_^2^ − 0.07X_3_^2^ + 0.12X_1_X_2_ − 0.02X_1_X_3_ + 0.01X_2_X_3_ (1)

The ANOVA analysis also allowed us to further confirm the reliability of the model. With high F-values and low associated *p*-values, the model was shown to be remarkably significant. The high R^2^ value (0.99) indicated that 99% of the variations are explained by the fitted model, while the adjusted determination coefficient (R^2^_Adj_) of 0.96, which is very close to R^2^, revealed a high degree of correlation between the observed and predicted values. Furthermore, the lack-of-fit test showed *p*-values much higher than 0.05, which means that the models can adequately fit to the experimental data. All these results indicate that the fitted model reliably explains the relation between the response and the independent variables and is suitable for predicting the response.

#### 2.2.2. Effect of the Independent Variables on the TPhC

**Figure 2:**
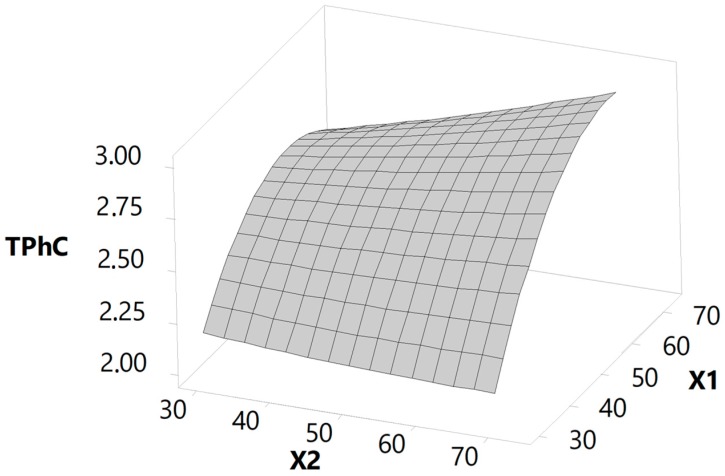
Response surface plots for the total phlorotannin content (TPhC in mg PGE/g DS) from *F. vesiculosus* extracts with respect to acetone concentration (%, X_1_) and solvent-solid ratio (mL/g, X_2_). The variable temperature was kept at its zero level.

The interactive effects of the significant terms, i.e., acetone concentration and solvent-solid ratio, on TPhC can be visualized on the three-dimensional response surface plot shown in Figure 2 which demonstrates the effects of these two independent variables on phlorotannin yields while the variable temperature is maintained at its zero level.

According to the graphical representation, it is unequivocal that acetone was the variable that most affected the TPhC values, with the maximum yield being achieved for a concentration of 67.2%. A slight decay of the phlorotannin content is observable when the acetone concentration increases up to 70%, particularly for low solvent-solid ratios, thus evidencing the quadratic effect that this variable has on this response. This confirms that the presence of water is important to confer a moderate polar medium to acetone that facilitates the extraction of hydrophilic phenolic compounds, which is the case of phlorotannins [44]. The variable solvent-solid ratio exhibited a directly proportional effect on the extracts’ TPhC, i.e., the higher the ratio, the greater the phlorotannin extraction from *F. vesiculosus*, reaching their maximum at 70 mL/g. This observation is however not in agreement with the single-factor experiments, since the total phlorotannin content of *F. vesiculosus* extracts were constant for ratios between 30 and 50 mL/g and decreased for ratios above (Figure 1C). This difference might be explained by the interactive effects that this variable has with the acetone concentration, which are very perceptible in the Figure 2, and which were not considered in the single-factor experiments. According to this picture, for high acetone concentrations, the response undergoes a great impact from the solvent-solid ratio variations, whereas for low acetone concentrations, the effect of this variable on TPhC is very tenuous. Consistent with these findings, other authors demonstrated that the extraction of phlorotannins from different brown seaweeds is greatly influenced by the solvent concentration or solvent-solid ratio [32,45], but none have yet shown the importance of the interaction between these two variables. Interestingly, although the single-factor experiments revealed that temperature significantly affects phlorotannin extraction from *F. vesiculosus* this was not observed in the experimental design, suggesting that the extraction of phlorotannins may be carried out at the most convenient temperature within the interval tested without affecting significantly the extraction yields.

#### 2.2.3. Optimization and Validation of the Models

Using the predictive equation mentioned above, the optimum conditions for the extraction of phlorotannins from *F. vesiculosus* were predicted as follows: acetone concentration at 67% (v/v), solvent-solid ratio at 70 mL/g, and temperature at 25 °C. Under these conditions the model predicted that the maximum phlorotannin content in the extracts would be 2.97 mg PGE/g DS (Table 2). 

To validate the reliability of the model, verification experiments were carried out three times under the predicted optimum parameters, and the experimental value of 2.92 ± 0.05 mg PGE/g DS was obtained. The good correlation that was observed between the experimental and predicted value confirms that this model is reliable and accurate.

### 2.3. Total Phlorotannin Content of the F. vesiculosus Extract and Respective Fractions

The crude extract obtained under the established optimum conditions was sequentially separated into three fractions, namely *n*-hexane (Hex) and ethyl acetate soluble (EtOAc) fractions and the final aqueous residue (AQ), by liquid−liquid partitioning. The differences in the extraction yields and total phlorotannin content between the crude extract and its subsequent fractions are depicted in Table 3.

A 28.2 ± 2.1% yield was obtained for the crude extract of *F. vesiculosus*, a value that is slightly higher than that described by Wang et al. [46] (20.2% w/w), and almost twice the yield reported by Liu et al. [25] (14.7% w/w), using both the same species and solvent. On the other hand, the total TPhC of the extract herein prepared was 10.7 ± 1.5 mg PGE/g extract, which is approximately 35 and 4 times lower compared to the values reported by those authors, respectively. Among several factors, this variability might be related to the geographical origin and/or harvest season of the algal material, as well as the methodology used to quantify the total phlorotannin content, since in the work of Wang et al. [46] the Folin-Ciocalteu was used instead of 2,4-dimethoxybenzaldehyde DMBA and only the latter is selective for phlorotannins [47]. After solvent partitioning, about 15.5%, 3.9% and 82.2% of the extract was distributed in the *n*-hexane, EtOAc and AQ fractions, respectively. The highest level of TPhC was found in the EtOAc with a value of 17.1 ± 1.5 mg PGE/g of dry residue, while Hex and AQ fractions showed the lowest TPhC both presenting approximately 4 mg PGE/g of dry residue. The use of ethyl acetate to selectively extract phlorotannins from various algae extracts has been a common practice [1]. In this work it is shown that this solvent can be used for concentrating/enriching phlorotannins from *F. vesiculosus* crude extract as well, which is in agreement with previous studies conducted in this species [25,46].

### 2.4. Inhibition of Enzymatic Activities

α-Amylase, α-glucosidase and pancreatic lipase are key enzymes in the digestive system, catalyzing the hydrolysis of complex food ingredients such as carbohydrates and triacylglycerols into simple and easily absorbable molecules. Inhibition of these enzymes therefore promotes a delay in the carbohydrate and lipid digestion and a consequent reduction of postprandial plasma glucose levels and overall bodyweight, thus contributing to the amelioration of type 2 diabetes mellitus and obesity symptoms. 

As shown in Table 4, the highest inhibitory activity of both crude extract and EtOAc was observed against α-glucosidase, followed by α-amylase and pancreatic lipase. Notably, the inhibitory capacities of crude extract and EtOAc against α-glucosidase were respectively 45 and 250 times stronger than that of acarbose (206.6 ± 25.1 μg/mL), the latter being a medication for type 2 diabetes mellitus. Interestingly, a significant increment of the inhibitory activity was noticed compared with previous data from our research group, in which the 70% acetone extracts of *F. vesiculosus* showed an IC_50_ of 32 μg/mL, which is likely to be related to factors such as the differences in the harvesting batch, as well as the differences in the extraction conditions [18]. EtOAc also exhibited better inhibitory activity than crude extract against α-amylase (2.8 ± 0.3 and 28.8 ± 1.2 μg/mL, respectively) and close to that of acarbose (0.7 ± 0.2 μg/mL), which is coherent with its higher content in phlorotannins. 

Likewise, although the effects of these samples against pancreatic lipase were far from matching that of orlistat (1.8 ± 0.5 ng/mL), an identical tendency was observed, i.e., EtOAc, which is higher in phlorotannins, presented better inhibitory activity than the crude extract (19.0 ± 1.8 and 45.9 ± 3.4 μg/mL, respectively). In fact, this outcome was already expected since phenolic compounds, particularly those with polymeric arrangements such as tannins, are well-known for their capacity to interact with proteins and form soluble or insoluble complexes [48]. Moreover, previous studies have already indicated that phlorotannin-rich extracts from *Fucus vesiculosus* and other species from the same genus are generally good inhibitors of α-glucosidase and α-amylase [16,49]. Interestingly, the inhibitions of approximately 470 and 305 times stronger than acarbose have been reported for *F. vesiculosus* aqueous and hydroethanolic extracts, respectively, against α-glucosidase, which is substantially higher than those herein reported for the EtOAc [17]. In this case, the inhibitory effects might be resultant from other non-phenolic compounds such as fucoidans and sulfated polysaccharides, which have been previously reported to display promising inhibitory activity against α-glucosidase [50]. In turn, the inhibitory activity of those extracts against α-amylase showed IC_50_ values of 63.5 and 59.1 μg/mL (for aqueous and hydroethanolic extracts, respectively), corresponding to twice or more the values herein observed [17]. 

To our knowledge, in addition to our previous works, only Chater et al. [19], reported the inhibitory activity of *Fucus* against pancreatic lipase. According to their study, from the four different *F. vesiculosus* preparations tested, namely homogenate, ethanol extract, ethanol pellet and sodium carbonate extract, good pancreatic lipase inhibitory effects were observed only for the first two (IC_50_ = 0.119 and 0.159 mg/mL, respectively), while sodium carbonate extract only presented an IC_50_ of approximately 1 mg/mL. Interestingly, previously results from our research group, showed that several *F. vesiculosus* ethanol extracts did not exert any inhibition of pancreatic lipase and only mild inhibition was observed for 70% acetone and aqueous extracts [18]. Once again, differences in the extraction procedures and experimental protocols might be the explanation for the observation of such discrepancies, as well as other non-considered factors.

Overall, these results suggest that phlorotannins from *F. vesiculosus* are important contributors to the antidiabetic and anti-obesity properties claimed for this species and that these compounds hold the potential to control blood glucose levels and overall energy intake through inhibition of α-glucosidase, α-amylase and pancreatic lipase. In fact, the active doses herein reported may be within physiological range to affect these three enzymes at a digestive level, since their action takes place in the digestive tract and, therefore, they are more exposed to phlorotannin interactions and less dependent of bioavailability-related issues. Hence, in addition to the possible medical and/or pharmaceutical applications, *F. vesiculosus* and its phlorotannins may also hold potential to be used as ingredients for the development of functional foods and even be consumed as functional food itself.

### 2.5. Characterization of Phlorotannin-Rich Fraction

**Figure 3:**
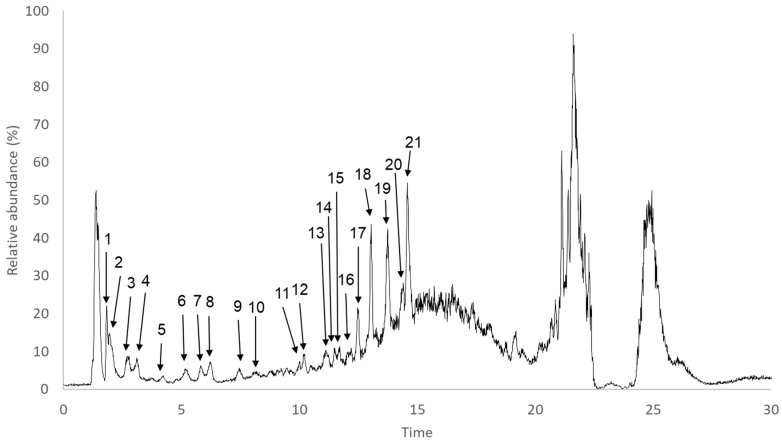
Total ion chromatogram (TIC) of the EtOAc fraction. Peaks marked with numbers correspond to the tentatively identified compounds represented in Table 5.

The presence of phlorotannins in brown seaweeds is widely acknowledged, although, due to their structural complexity and similarity, as well as the lack of commercial standards, the identification and characterization of these compounds is usually a challenging task. Due to higher TPhC and anti-enzymatic activity, the UHPLC-MS analysis was carried out using the EtOAc fraction. The total ion chromatogram (Figure 3) obtained was characterized by a region of reasonably well separated peaks (up to 10 min), corresponding to phlorotannin oligomers, and another region where higher polymeric phlorotannin structures are eluted together in a hump [22,51]. Overall, a total of 21 peaks were analyzed, all exhibiting a UV-max around 270 nm, which is in line with what has been reported in the existing literature on phlorotannins [52] and is very close to that of phloroglucinol (267 nm). Moreover, in almost every identified compound, the base peak at MS^2^ corresponded to the loss of one or two water molecules, which is also a common characteristic in these compounds. The tentative identification of these compounds was further carried out based on an MS^2^ fragmentation pattern as well as by comparison with data previously reported in the literature.

**Figure 4:**
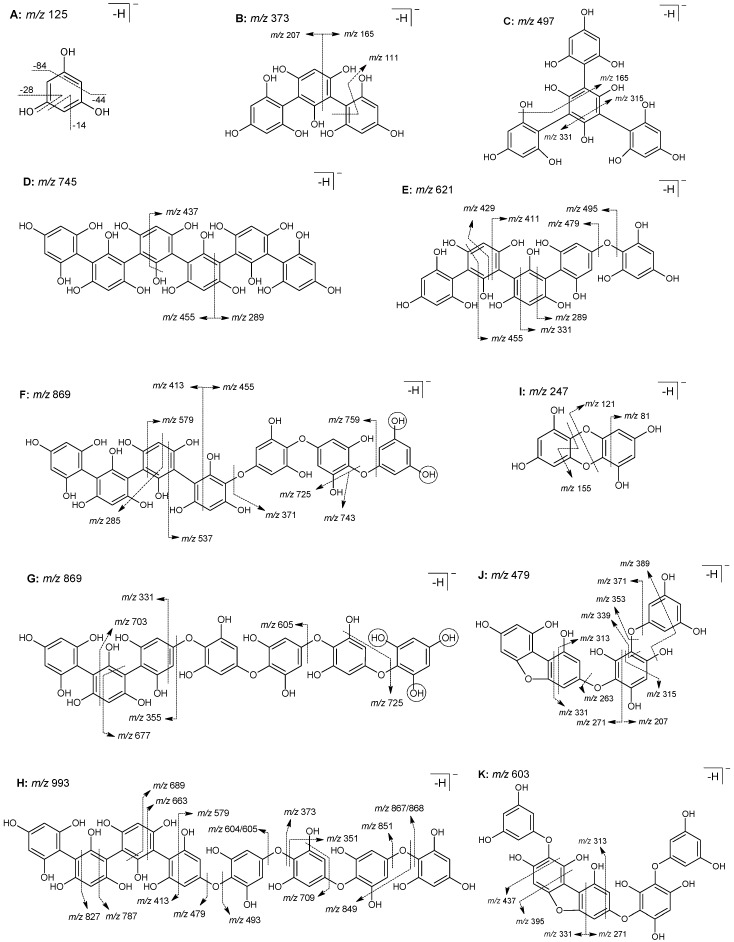
Structure of phlorotannin compounds tentatively identified in *F. vesiculosus* EtOAc fraction and proposed fragmentation patterns: (**A**) phloroglucinol ([M − H]^−^ at *m/z* 125), (**B**) bifucol ([M − H]^−^ at *m/z* 373), (**C**) trifucol ([M − H]^−^ at *m/z* 497), (**D**) hexafucol ([M − H]^−^ at *m/z* 745), (**E**) ([M − H]^−^ at *m/z* 621), (**F**) trifucodiphlorethol ([M − H]^−^ at *m/z* 869), (**G**) difucotriphlorethol ([M − H]^−^ at *m/z* 869), (**H**) trifucotetraphlorethol ([M − H]^−^ at *m/z* 993), (**I**) dibenzodioxine-1,3,6,8-tetraol ([M − H]^−^ at *m/z* 247), (**J**) fucofurodiphlorethol ([M − H]^−^ at *m/z* 479) ([M − H]^−^ at *m/z* 603). Fragmentations with simultaneous loss of water are only representative. Cleavage of the OH group may occur at different sites.

Several types of phlorotannins could be noted in this fraction, including fucols, fuhalols and fucophlorethols. Fucols consist of polymers of phloroglucinol (A in Figure 4) linked together through C−C bonds, while phlorethols are polymers of the same compound linked through C−O−C linkages. This is a relevant detail when it comes to the identification of these compounds through MS, since C−C bonds usually require higher energy to break than C−O−C linkages, and therefore, although fucols, phlorethols and fucophlorethols with the same degree of polymerization will have the same molecular weight, they can produce different fragmentation patterns [53]. In turn, fuhalols are ether-linked phloroglucinol units that contain at least one additional hydroxyl group [38].

Based on MS/MS analysis as well as data reported in literature, three compounds were tentatively identified as belonging to the group of fucols and, despite the fact that their exact structural features were not possible to disclose, these consisted of trifucol ([M − H]^−^ at *m/z* 373, peak 1), tetrafucol ([M − H]^−^ at *m/z* 497, peak 2) and hexafucol ([M − H]^−^ at *m/z* 745, peak 5). Overall, the MS^2^ spectrum of these compounds exhibited common losses of 14, 44 and 84 Da (as depicted in structure A of Figure 4), and/or their combinations with water (60 and 102 Da, respectively), phloroglucinol units (e.g., 166 and 208 Da, respectively) or water plus phloroglucinol units, which are indicative of cross-ring cleavages (Table 4, B–D in Figure 4), while product ions resulting from the loss of phloroglucinol moieties were present in very low intensities or even completely absent. Note that to our knowledge, this type of fragment has rarely been described in previous studies on the interpretation of MS product ions aiming at phlorotannin identification. In fact, although losses of 44, 28 or 14 Da (and their derivatives) have been previously assigned in these phenolic compounds [46,53], other possible cross-ring cleavages are being herein elucidated for the first time.

In contrast, the compounds with [M − H]^−^ at *m/z* 621, eluting in peak 3, [M − H]^−^ at *m/z* 869, eluting in peaks 6 and 7, and [M − H]^−^ at *m/z* 993, eluting in peaks 9, 10, 11 and 12, were identified as fucophlorethols since their MS^2^ spectra presented product ions resultant from cross-ring cleavages, and simultaneously, several originated from the loss of one or more phloroglucinol moieties (−124/126 Da): *O*-phloroglucinol (−140/142) and its fuhalol derivatives and/or resorcinol (108/110), which are indicative of ether bond cleavages [52]. The identification of the correct structure of these compounds through LC-MS is, however, very difficult, particularly for those with high molecular weights, since linkage positions cannot be assigned. Therefore, note that structures E–L in Figure 4 are representative of compounds that may occur in different isomeric forms. In this context, the compound with [M − H]^−^ at *m/z* 621 exhibited two product ions at *m/z* 495 and 479 (corresponding to the loss of phloroglucinol and *O*-phloroglucinol moieties, respectively) in its MS^2^ spectrum, thus suggesting a fucophlorethol pentamer structure with at least one ether bond in its backbone, as represented in E from Figure 4. In turn, the compounds with [M − H]^−^ at *m/z* 869 presented slightly different fragmentation patterns. As detailed in structure F from Figure 4, the MS^2^ spectrum of the one eluting in peak 6 exhibited product ions at *m/z* 743 (−126 Da), 725 (−126–18 Da), 759 (−110 Da) and 371 (−498 Da), corresponding to the loss of a phloroglucinol, a phloroglucinol plus water, a resorcinol and four phloroglucinol units, respectively, whereas the compound eluted in peak 7 (G in Figure 4) showed the product ions at *m/z* 725 (loss of 1 PGU plus water), *m/z* 605 (loss of bifuhalol) and at *m/z* 355 (loss of tetrafuhalol). In these compounds, the losses of resorcinol or bifuhalol moieties are particularly relevant for unveiling additional information about their structural arrangement. The former indicates that the terminal phloroglucinol unit contains two free OH groups (see highlights in the compound F from Figure 4), while the latter indicates that the terminal phloroglucinol unit contains three free OH groups (see highlights in the structure G from Figure 4). Based on these MS data, it is possible to conclude these two compounds are both fucophlorethol heptamers, containing at least two and three C−O−C bonds in their backbones, respectively.

The fragmentation patterns among the four compounds with [M − H]^−^ at *m/z* 993 also revealed some differences that evidenced their structural diversity. Yielding the product ions at *m/z* 867 (loss of phloroglucinol), *m/z* 849 (loss of phloroglucinol plus water) and *m/z* 709 (loss of bifuhalol plus water), it is clear that the nonamer eluting in peak 9 contains at least 3 C−O−C linkages, while based on the MS fragmentation pattern of the compound eluting in peak 10 it is only possible to confirm the presence of one ether linkage (i.e., only *m/z* at 867 was observed). In turn, the fragmentation pattern of the compound eluting in peak 11 showed the product ions at *m/z* 867, 849, 831 (loss of PGU, PGU plus water and PGU plus 2 waters, respectively), 604 (loss of trifuhalol + 1), 479 (loss of tetrafuhalol) and 373 (loss of 5 PGUs) as represented in H from Figure 4, thus suggesting the presence of at least four ether linkages in the backbone of this isomer. Interestingly, even though MS^2^ spectra from the compound eluting in peak 12 also indicate the presence of four ether linkages, this compound produced a different fragmentation pattern, yielding fragment ions at *m/z* 851 (loss of *O*-phloroglucinol), 605 (loss of trifuhalol), 493 (loss of 4 PGUs + 2) and 351 (loss of 5 PGUs plus water + 2), thus evidencing structural dissemblance compared with the previous one. Note that despite fucophloretols of variable DP having been already documented in *F. vesiculosus*, the examination of their structural features is not commonly approached. In fact, to our knowledge, a detailed scrutinization of structural features of *F. vesiculosus* phlorotannin compounds by HPLC-MS has only been carried out by Lopes et al., for those with DP below 6 units [53].

Other compounds with more than 8 phloroglucinol units have also been detected in this *F. vesiculosus* fraction, although due to their low intensities in the MS spectra and the impossibility to go on with further fragmentation in tandem MS, additional structural details of these compounds were not obtained. Nevertheless, some deprotonated molecular ions at *m/z* 1117 and 1241, consistent with the molecular weight of phlorotannin nonamers and decamers, respectively [51], were found co-eluting in peaks 13, 14 and 15 (data not shown). Moreover, several minor compounds co-eluting in the peaks 11–17 showed deprotonated molecular ions at *m/z* 806, 868, 930, 992, 1116, 1054, 1178, 1240, 1302 and 1364 (data not shown) which are consistent with the doubly charged ions of phlorotannins with DP 13–22, respectively [54]. Notably, detection of compounds with such polymerization degrees in Portuguese-sourced *F. vesiculosus* has not been achieved in the study of Lopes et al., although Steevensz et al. [54] have already described the presence of phlorotannin polymers with up to 39 units in samples of the same species, collected in Nova Scotia, Canada.

The group of identified fuhalols comprised a hydroxytetrafuhalol ([M − H]^−^ at *m/z* 529) and two isomers of pentafuhalol ([M − H]^−^ at *m/z* 637), which, in addition to the evident extra OH groups, showed MS/MS fragmentation patterns concordant to previous data reported in the literature [55]. Notably, although the former has been described in *F. vesiculosus* before, pentafuhalol had not been detected yet in the genus *Fucus* [56].

Apart from the fucols, fucophlorethols and fuhalols, other unusual phlorotannin compounds were identified in this *F. vesiculosus* fraction as well. This is the case of the phloroglucinol dimmer with *m/z* at 247 eluting in peak 3. The MS/MS of this compound produced the main product ion at *m/z* 203 ([M − H − 44]^−^) and ions at *m/z* 121, 81 and 155, corresponding to the [M − H − PGU]^−^, [M − H − dihydroxybenzodioxin]^−^ and a methoxy-phloroglucinol moiety, respectively, suggesting the presence of dibenzodioxine-1,3,6,8-tetraol, i.e., a precursor of an eckol type phlorotannin (I in Figure 4). Interestingly, small phlorotannins, in particular those with DP 2, are not commonly described in this species. In fact, *Fucus* are usually described to be more abundant in phlorotannin oligomers of higher DPs (5–10 units) [51,54]. Moreover, to our knowledge, this compound has only been described once in *A. nodosum* extracts among Fucaceae [57].

Additionally, two uncommon molecular ions were found at *m/z* 479 (eluting in peaks 7 and 8) and 603 (eluting in peak 9). The MS spectrum of the former suggests two isomers of a dehydroxylated phlorotannin tetramer both further producing atypical fragments at *m/z* 271 (PGU-84 Da) as well as an atypical loss of 148 Da that were not visible in the other tetramers and indicate the presence of a furan ring (J in Figure 4). Likewise, [M − H]^−^ at *m/z* 603 resembles a pentamer lacking an OH group, and also presented fragments ([M − H]^−^ at *m/z* 271 and 315) that suggest the presence of a furan ring as well (K in Figure 4). Based on their MS spectra and the published literature [1], it is possible that the structures of these tetramers and pentamer resemble that of fucofuroeckol ([M − H]^−^ at *m/z* 477) and phlorofucofuroeckol ([M − H]^−^ at *m/z* 601), respectively, although with an ether linkage instead of a dioxin ring between the two inner phloroglucinol moieties, which would explain the 2 Da difference between their deprotonated ions. Previous works have already reported fucofuroeckol derivatives in the genus *Fucus* [53], however, such compounds have never been described before, thus a proper purification and isolation of these compounds would be necessary to allow further spectroscopic analysis, such as NMR, aiming the better elucidation of their structural features. Following the same logic, it is possible that the [M − H]^−^ at *m/z* 851 (eluting in peak 14) belongs to the same group of compounds, containing two additional phloroglucinol units, although there is no previous literature describing resembling structures.

Notably, even though their structural elucidation were not achieved, many other compounds were identified as phlorotannin derivatives (Table 5) based on their MS/MS spectra which exhibited fragmentation patterns similar to those of phlorotannin compounds, either yielding product ions that are indicative of one or multiple phloroglucinol units (e.g., *m/z* 125, 247/249, 371, 495/497, 621), or fragments that are indicative of phloroglucinol or phloroglucinol derivative losses (e.g., −124/126, −140/142, −144, −166, −248/250, −266, −374), as well as the usual water losses or cross ring cleavages.

## 3. Materials and Methods

### 3.1. Materials

Grounded *F. vesiculosus* samples from July 2017 were purchased from Algaplus Lda (production site located at Ria de Aveiro coastal lagoon, Northern Portugal, 40°36′43″ N, 8°40′43″ W) an enterprise dedicated to the production of edible seaweeds in an integrated multi-trophic aquaculture (IMTA) system. HPLC grade acetone, ethanol, methanol, *n*-hexane, ethyl acetate, acetonitrile, dimethylsulfoxide, hydrochloric acid, glacial acetic acid, sodium chloride, sodium hydroxide, potassium hydroxide, sodium and potassium tartarate, tris-HCl, and starch were acquired from Fisher (Pittsburgh, PA, USA). The enzymes α-glucosidase from *Saccharomyces cerevisiae* (EC No. – 3.2.1.20), α-amylase from porcine pancreas (EC No. – 3.2.1.1) and lipase from porcine pancreas (EC No. – 3.1.1.3) together with 2,4-dimethoxybenzaldehyde (DMBA), 4-nitrophenyl α-D-glucopyranoside (PNPG), 4-nitrophenyl butyrate (PNPB) and formic acid were purchased from Sigma (St. Louis, MO, USA). Sodium di-hydrogen phosphate and potassium di-hydrogen phosphate were acquired from Panreac (Barcelona, Spain). Dinitrosalycilic acid and Acarbose were purchased from Acros Organics (Hampton, NH, USA), calcium chloride from ChemLab (Eernegem, Belgium) and orlistat from AlfaAesar (Ward Hill, MA, USA). All reagents were of analytical grade or of the highest available purity.

### 3.2. Methods

#### 3.2.1. Single-Factor Experiments

Extractions were performed using the conventional mechanical stirring solid-liquid method at atmospheric pressure. For each experiment, 1 g of dried *F. vesiculosus* powder was loaded into glass flasks covered with aluminum foil. Initial extraction conditions were as described by Neto et al. [18] and the experiments were carried by varying one condition at a time, namely solvent concentration (10–90% v/v), temperature (25–50 °C), solvent-solid ratio (10–110 mL/g) and extraction time (1–9 h). The flasks were all screw capped to control solvent evaporation and kept under constant agitation. Finally, the extracts were centrifuged at 6000 rpm at 4 °C for 10 min and the supernatant was filtered and stored at –20 °C until subsequent use.

#### 3.2.2. Experimental Design for Optimization of Phlorotannins Extraction 

A three level, three-variable Box–Behnken experimental design (BBD) was employed in this study for evaluating the effects of solvent concentration (% v/v, *X*_1_), solvent-solid ratio (mL/g, *X*_2_) and extraction time (h, *X*_3_) on the total phlorotannin content (TPhC, mg PGE/g dry seaweed) of *F. vesiculosus*. The levels of these three variables (Table 6) were set according to the single-factor tests outlined above.

A total of 15 different experiments, including three replicates at central point (Table 7), were conducted in a randomized order. Using the response surface methodology, the experimental design and analysis of variance (ANOVA) were carried out in the statistical software JMP, version 10.0.0 (Cary, NC, USA), to generate the following second-order polynomial equation that represents the total phlorotannin content as a function of the coded independent variables:

(2) $$Y=\beta_{0}+\overset{k}{\underset{i=1}{\sum }}\beta_{i}X_{i}+\overset{k}{\underset{i=1}{\sum }}\beta_{ii}X_{i}^{2}+\overset{k}{\underset{i\neq j=1}{\sum }}\beta_{ij}X_{i}X_{j}$$

where *Y* is the predicted response; β_0_ is the constant coefficient; β_i_, β_ii_ and β_ij_ are the linear, quadratic and interactive coefficients of the model, respectively; and *X*_i_ and *X*_j_ are the coded independent variables. 

Model adequacy was evaluated using the coefficient of determination (R^2^) and the lack-of-fit test represented at 5% level of significance, accordingly. Three-dimensional response surface plots and two-dimensional contour plots were used for visualization of the effects of independent variables and their mutual interactions in the response. To validate the accuracy of the model, triplicate experiments were carried out at the optimal conditions predicted for TPhC, and the obtained experimental data were compared to the values predicted by the regression model.

#### 3.2.3. Preparation and Purification of Seaweed Extract

The solvent extracts were prepared following the optimum conditions determined through the response surface method. Briefly, 30 g of dried algal powder were dispersed in 2100 mL of 70% acetone solution and incubated in for 3 h at room temperature under constant agitation. The mixture was filtered through cotton to remove the solid residues and then through a G4 glass filter. Afterwards the extract was concentrated in a rotary evaporator to about 250 mL. Concentrated extract was defatted using *n*-hexane (1:1, v/v) several times until a colorless nonpolar fraction was obtained, and the aqueous phase was further submitted to liquid-liquid extraction with ethyl acetate (1:1, v/v) three times, to obtain a phlorotannin-purified fraction. Finally, the solvents from the three fractions, including aqueous residue, were removed by rotary evaporation and subsequently stored at −20 °C until further analysis.

#### 3.2.4. Determination of Total Phlorotannin Content

Quantification of total phlorotannins was carried out according to the 2,4-dimethoxybenzaldehyde (DMBA) colorimetric method previously described [58]. Briefly, equal volumes of the stock solutions of DMBA (2%, m/v) and HCl (6%, v/v), both prepared in glacial acetic acid, were mixed prior to use (work solution). Afterwards, 250 μL of this solution was added to 50 μL of each extract in a 96-wells plate and the reaction was incubated in the dark, at room temperature. After 60 min, the absorbance was read at 515 nm and the phlorotannin content was determined by using a regression equation of the phloroglucinol linear calibration curve (0.06–0.1 mg/mL). The results were expressed as mg phloroglucinol equivalents/g dry seaweed (mg PGE/g DS).

#### 3.2.5. Enzymatic Assays

##### α-amylase Inhibition Assay 

Inhibition of α-amylase activity was measured according to Pereira et al. [59], with slight modifications. Briefly, 200 µL of extract six different extract concentrations (0–0.06 mg/mL for crude extract and 0–0.005 mg/mL for EtOAc fraction) dissolved in 20 mM phosphate buffer (pH 6.9, containing 6 mM of NaCl) were added to 400 µL of a 0.8% (w/v) starch solution in the same phosphate buffer and the mixture was incubated for 5 min at 37 °C. The reaction was then started with the addition of 200 µL of α-amylase solution and after 5 min of incubation, 200 µL of the reaction mixture were collected and immediately mixed with 600 µL of DNS reagent (10 g/L of 3,5-dinitrosalicylic acid, 300 g/L of potassium and sodium tartrate tetrahydrate and 0.4 M NaOH) to stop the reaction. A second aliquot of 200 µL was further collected 15 min later and mixed with DNS reagent as well. Samples were then boiled for 10 min and once they had cooled, 250 µL were transferred to the wells in a 96-well microplate for absorbance reading at 450 nm. Blank readings (no enzyme) were then subtracted from each well and the inhibitory effects towards α-amylase activity was calculated as follows:

(3) $$\%\;\mathrm{inhibiton}=\frac{\Delta {\mathrm{Abs}}_{c}-\Delta {\mathrm{Abs}}_{e}}{\Delta {\mathrm{Abs}}_{c}}\times 100$$

where ΔAbs_c_ is the variation in the absorbance of the negative control and ΔAbs_e_ is the variation in the absorbance of the extract. Acarbose was used as a positive control of inhibition.

##### α-glucosidase Inhibition Assay 

Inhibition of α-glucosidase was measured according to the method previously described by Neto et al. [18]. In short, 50 µL of different extract concentrations (0–0.006 mg/mL for crude extract and 0–0.001 mg/mL for EtOAc fraction, in 50 mM phosphate buffer pH 6.8) were mixed with 50 µL of 6 mM 4-nitrophenyl α-D-glucopyranoside (pNPG), dissolved in deionized water. The reaction was started with the addition of 100 µL of α-glucosidase solution and the absorbance was monitored at 405 nm every 60 s for 20 min at 37 °C. Acarbose was used as positive control of inhibition.

##### Pancreatic Lipase Inhibition Assay 

Inhibition of lipase activity was measured following the procedure described by Olivia et al. [59]. The reaction mixture was prepared in a microtube by mixing 55 µL of five different concentrations of extract (0–0.4 mg/mL for crude extract and 0–0.07 mg/mL for EtOAc fraction) dissolved in Tris buffer 100 mM (pH 7.0) with 467.5 µL of Tris-HCl (100 mM, pH 7.0, containing 5 mM of CaCl_2_) and 16.5 µL of enzyme. The reaction was started by adding 11 µL of 20 mM 4-nitrophenyl butyrate diluted in DMSO. The final DMSO concentration in the reaction mixture did not exceed 2%. The reaction mixture was then quickly transferred to a 96-well plate and incubated for 35 min at 37 °C while the absorbance was being measured every 60 s at 410 nm. Orlistat was used as a positive control of inhibition.

#### 3.2.6. UHPLC-DAD-ESI/MS Analysis

Chromatographic analysis of *F. vesiculosus* phlorotannin-enriched ethyl acetate fraction was carried out in Ultimate 3000 (Dionex Co., San Jose, CA, USA) an apparatus consisting of an autosampler/injector, a binary pump, a column compartment and an ultimate 3000 Diode Array Detector (Dionex Co.,San Jose, CA, USA), coupled to a Thermo LTQ XL (Thermo Scientific, San Jose, CA, USA) ion trap mass spectrometer equipped with an ESI source. The LC separation was conducted using a method adapted from Ferreres et al. [60] with a Hypersil Gold (ThermoScientific, San Jose, CA, USA) C18 column (100 mm length; 2.1 mm i.d.; 1.9 μm particle diameter, end-capped) maintained at 30 °C and a binary solvent system composed (A) acetonitrile and (B) 0.1% of formic acid (v/v). The solvent gradient started with 5–40% of solvent (A) over 14.72 min, from 40–100% over 1.91 min, remaining at 100% for 2.19 more min before returning to the initial conditions. The flow rate was 0.2 mL min^−1^ and UV–Vis spectral data for all peaks were accumulated in the range of 200–700 nm while the chromatographic profiles were recorded at 280 nm. Control and data acquisition of MS were carried out with the Thermo Xcalibur Qual Browser data system (ThermoScientific, San Jose, CA, USA). Nitrogen above 99% purity was used, and the gas pressure was 520 kPa (75 psi). The instrument was operated in negative mode with the ESI needle voltage set at 5.00 kV and an ESI capillary temperature of 275 °C. The full scan covered the mass range from *m/z* 100 to 2000. CID–MS/MS experiments were performed for precursor ions using helium as the collision gas with a collision energy of 25–35 arbitrary units. All solvents used were of LC-MS grade.

#### 3.2.7. Statistical Analysis

All data were expressed as mean ± standard error of the mean (SEM) of three similar and independent experiments performed in duplicate. JMP, version 10.0.0 (Cary, NC, USA) and Minitab, version 17.3.1. (Paris, France) softwares were used to construct the BBD and to analyze the results. Data from single-factor experiments and BBD were analyzed using ANOVA (*p* < 0.05) followed by Tukey’s post hoc test.

## 4. Conclusions

In the present study, a single-factor experimental approach followed by a response surface methodology were carried out for determination of the optimum conditions that maximize the extraction of phlorotannins from *Fucus vesiculosus*. The optimal extraction conditions established were: X_1_ = 67% (v/v), X_2_ = 70 mL/g and X_3_ = 25 °C. Under the optimized conditions, the experimental values agreed with the values predicted by each regression equation, allowing the validation of the accuracy and predictive capacity of the model. The optimized extract showed promising inhibitory effects against pancreatic lipase, α-amylase and particularly α-glucosidase. Improvement of the anti-enzymatic inhibitory effects were observed in the phlorotannin-purified fraction, particularly towards α-amylase, which is coherent with its higher content in phlorotannins. UHPLC-DAD-ESI-MS^n^ analysis of this purified fraction allowed us to disclose new structural features of *F. vesiculosus* phlorotannins through a detailed interpretation of their fragmentation patterns. Overall, this purified extract was composed of fucols, fucophlorethols, fuhalols together with several other phlorotannin derivatives of variable degrees of polymerization, ranging from 3 to 22 phloroglucinol units. Additionally, the appearance of possible new phlorotannin compounds, tentatively identified as fucofurodiphlorethol, fucofurotriphlorethol and fucofuropentaphlorethol was also observed in this extract of *F. vesiculosus*, although further research is necessary to verify their structural arrangements. 

Overall, this work contributes valuable insights on the phlorotannin profile of *F. vesiculosus* through mass spectrometry, simultaneously supporting the potential of this seaweed to be exploited as a valuable source of phlorotannins for commercial applications as novel functional ingredients for development of functional foods, nutraceuticals or even pharmaceutical products targeting diabetes and obesity prevention. 

## Figures and Tables

**Table 1:** Regression coefficients and results of ANOVA analysis of the model.

Parameter	Regression Coefficient
**β_0_**	2.69 ***
***X*_1_**	0.31 ***
***X*_2_**	0.08 **
***X*_3_**	0.02
***X*_1_*X*_1_**	−0.25 ***
***X*_2_*X*_2_**	0.02
***X*_3_*X*_3_**	−0.07
***X*_1_*X*_2_**	0.12 **
***X*_1_*X*_3_**	−0.02
***X*_2_*X*_3_**	0.01
**R^2^**	0.99
**R^2^_Adj_**	0.96
**Model F-value**	39.24
**Model *p*-value**	<0.001
**Lack-of-fit *p*-value**	0.46

β_0_—constant coefficient; *X*_1_—acetone concentration (%); *X*_2_—solvent-solid ratio (mL/g); *X*_3_—extraction temperature (°C). **, *** represent statistical significance with *p* < 0.01 and 0.001, respectively.

**Table 2:** Predicted and experimental values obtained for TPhC according to the predicted optimum conditions.

Response	Optimum Conditions			Results	
	*X*_1_	*X*_2_	*X*_3_	Predicted	Experimental
TPhC (mg PGE/g DS)	67	70	25	2.97	2.92 ± 0.05

*X*_1_—acetone concentration (%, v/v); *X*_2_—solvent-solid ratio (mL/g); *X*_3_—temperature (°C); TPhC—total phlorotannin content; PGE—phloroglucinol equivalents.

**Table 3:** Extraction yield (as w/w of algal powder for crude extract and w/w of crude extract for the fractions) and total phlorotannin content of *F. vesiculosus* crude extract and further fractions.

Sample	Yield (%)	TPhC (mg PGE/g ext)
Crude extract	28.2 ± 2.1	10.7 ± 1.5 ^b^
Hex	15.5 ± 1.2 ^b^	4.0 ± 0. 9 ^c^
EtOAc	3.9 ± 0.6 ^c^	17.1 ± 1.5 ^a^
AQ	82.2 ± 2.3 ^a^	3.7 ± 0.5 ^c^

PGE—phloroglucinol equivalents; ext—extract; Hex—*n*-hexane fraction; EtOAc—ethyl acetate fraction; AQ—aqueous residue. Data expressed as mean ± standard deviation. Different letters within a column mean significantly differences at *p* < 0.05 using student’s t test.

**Table 4:** Inhibition of α-glucosidase, α-amylase and lipase by *F. vesiculosus* crude extract, ethyl acetate fraction and the respective reference compounds.

Sample	IC_50_ Value (μg/mL)		
	α-amylase	α-glucosidase	Pancreatic Lipase
Crude extract	28.8 ± 1.2 ^a^	4.5 ± 0.7 ^a^	45.9 ± 3.4 ^a^
EtOAc	2.8 ± 0.3 ^b^	0.82 ± 0.05 ^a^	19.0 ± 1.8 ^b^
Acarbose	0.7 ± 0.2 ^c^	206.6 ± 25.1 ^b^	-
Orlistat *	-	-	1.8 ± 0.5 ^c^

EtOAc—ethyl acetate fraction from *F. vesiculosus* extract. IC_50_ value was determined as the concentration at which α-amylase, α-glucosidase, and pancreatic lipase were inhibited by 50%. All values are expressed as mean ± SD. Different letters within a column mean significantly differences at *p* < 0.05 using student’s t test. * IC_50_ value for orlistat is expressed in ng/mL.

**Table 5:** Tentative assignment of the compounds present in the ethyl acetate fraction of *F. vesiculosus* extract analyzed by LC-ESI-MS/MS.

Peak	RT (min)	[M − H]^−^ (*m/z*)	MS/MS Fragments (-loss) *	Tentative Assignment
1	1.8	373	MS^2^[373]: **355** (−18), 329 (−44), 207 (−166), 165 (−PGU−84), 289 (−84), 111 (−2PGU−14),	Trifucol
2	1.9	497	MS^2^[497]: **479** (−18), 331 (−166), 461 (−36), 453 (−44), 435 (−44−18), 395 (−84−18), 165 (−2PGU−84), 315 (−166−18), 413 (−84)	Tetrafucol
		529	MS^2^[529]: **511** (−18), 493 (−36), 467 (−44−18), 411 (−84−36, +2), 449 (−44−36), 485 (−44), 347 (−166−18, +2), 405 (−PGU), 377 (−PGU−28)	Hydroxytetrafuhalol
		689	MS^2^[689]: **605** (−84), 497 (−192), 621 (−68), 553 (−136), 671 (−18), 653 (−36), 537 (−PGU−28), 643 (−46), 575 (−114), 507 (−182), 345 (−2PGU−96)	Phlorotannin derivative
3	2.8	621	MS^2^[621]: **603** (−18), 455 (−166), 585 (−36), 331 (−PGU-166), 577 (−44), 559 (−44−18), 519 (−84−18), 289 (−2PGU−84), 429 (−192), 537 (−84), 495 (−PGU), 479 (−*O*–PGU), 411 (−PGU-84)	Trifucophlorethol
		247	MS^2^[247]: **202** (−45), 121 (−PGU), 81 (−166), 155 (−PGU-29)	Dibenzodioxine-1,3,6,8-tetraol
4	3.1	555	MS^2^[555]: **537** (−18), 511 (−44), 519 (−36), 389 (−166), 331 (−224), 363 (−192), 393 (−PGU−36), 413 (−*O*–PGU), 430 (−PGU, −1), 305 (−2PGU), 247 (−308), 223 (−2PGU-84), 165 (−trifuhalol)	Phlorotannin derivative
5	4.2	745	MS^2^[745]: **727** (−18), 455 (−PGU−166), 709 (−36), 579 (−166), 289 (−3PGU−84), 701 (−44), 683 (−44−18), 643 (−84−18) 437 (−PGU−166−18)	Hexafucol
6	5.2	623	MS^2^[623]: **495** (−110−18), 477 (−110−36), 605 (−18), 369 (−2PGU, −2), 249 (−3PGU)	Phlorotannin derivative
		869	MS^2^[869]: **851** (−18), 833 (−36), 743 (−PGU), 841 (−28), 725 (−PGU−18), 313 (−2PGU−166−18), 759 (−110), 413 (−2PGU−166), 579 (−PGU−166), 537 (−2PGU-84), 285 (−4PGU−72−18), 825 (−44), 455 (−3PGU−84), 371 (−4PGU)	Trifucotriphlorethol
7	5.8	869	MS^2^[869]: **833** (−36), 851 (−18), 703 (−166), 677 (−192), 767 (−84−18), 785 (−84), 725 (−PGU−18), 605 (−PGU-140), 355 (−tetrafuhalol), 331 (−3PGU−166)	Difucotetraphlorethol
		479	MS^2^[479]: **461** (−18), 435 (−44), 433 (−28−18), 389 (−72−18), 313 (−166), 315 (−164), 271 (−PGU−84), 443 (−36), 339 (−140), 371 (−108), 451 (−28), 207 (−272)	Fucofurodiphlorethol
8	6.2	479	MS^2^[479]: **461** (−18), 435 (−44), 433 (−28−18), 389 (−72−18), 315 (−164), 443 (−36), 371 (−108), 271 (−208), 331 (−148), 353 (−126), 451 (−28), 263 (−216)	Fucofurodiphlorethol
9	7.5	993	MS^2^[993]: **975** (−18), 965 (−28), 827 (−166), 849 (−PGU-18), 868 (−PGU, +1), 957 (−36), 413 (−4PGU-84), 709 (−bifuhalol−18)	Pentafucodiphlorethol
		603	MS^2^[603]: **585** (−18), 559 (−44), 437 (−166), 395 (−PGU−84), 313 (−PGU−166), 271 (−2PGU−84), 331 (−272)	Fucofurotriphlorethol
10	8.2	385	MS^2^[385]: **259** (−PGU), 367 (−18), 341 (−44), 245 (−140), 357(−28), 261 (−PGU), 313 (−72), 219 (−166)	Phlorotannin derivative
		993	MS^2^[993]: **975** (−18), 579 (−2PGU−166), 957 (−36), 827 (−166), 849 (−PGU-18), 909 (−84), 891 (−84−18), 867 (−PGU), 949 (−44), 413 (−4PGU−84)	Hexafucophlorethol
		623	MS^2^[623]: **605** (−18), 579 (−44), 495 (−110−18), 535 (−88), 561 (−44−18), 551 (−72), 357 (−bifuhalol), 437 (−PGU−44−18), 457 (−166)	Phlorotannin derivative
11	10.0	363	MS^2^[363]: **319** (−44), 345 (−18), 222 (−*O*–PGU, −1), 331 (−32), 301 (−44−18), 178 (−185), 327 (−36),	Phlorotannin derivative
		993	MS^2^[993]: **975** (−18), 965 (−28), 867 (−PGU), 604 (−trifuhalol, +1), 579 (−2PGU−166), 849 (−PGU−18), 479 (−tetrafuhalol), 831 (−PGU-36), 787 (−PGU-84, +2), 373 (−5PGU)	Tetrafucotetraphloretol
		771	MS^2^[771]: **753** (−18), 727 (−44), 735 (-36), 496 (−274, +1), 471 (−300), 477 (−294), 504 (−bifuhalol, +1), 615 (−156), 263 (−508), 587 (−184), 643 (−110−18), 613 (−158), 373 (−398)	Phlorotannin derivative
12	10.2	361	MS^2^[361]: **317** (−44), 343 (−18), 178 (-183), 331 (−30), 273 (−88), 299 (−44−18), 289 (−72)	Phlorotannin derivative
		993	MS^2^[993]: **975** (−18), 965 (−28), 957 (−36), 851 (−*O*–PGU), 493 (−4PGU, −2), 663 (−4PGU−166), 689 (−2PGU−56), 351 (−5PGU-18), 457 (−3PGU−84), 605 (−trifuhalol, +2)	Tetrafucotetraphloretol
13	11.1	403	MS^2^[403]: **261** (−*O*–PGU), 385 (−18), 259 (−44), 217 (−186), 327 (−76), 371 (−32), 309 (−94), 341 (−44−18), 353 (−50), 193 (−PGU−84), 141 (−262), 125 (−278)	Phlorotannin derivative
		711	MS^2^[711]: **693** (−18), 623 (−88), 229 (−482), 563 (−148), 429 (−282), 579 (−132), 249 (−462)	Phlorotannin derivative
		637	MS^2^[637]: **619** (−18), 496 (−141), 511 (−126), 335 (−248−54), 593 (−44), 575 (−62) 436 (−182−17), 371 (−266), 601 (−36), 261 (−374−18), 245 (−266−126)	Pentafuhalol
14	11.5	317	MS^2^[317]: **273** (−44), 176 (−141), 299 (−18), 255 (−44−18), 245 (−72), 229 (−88), 187 (−130), 124 (−193)	Phlorotannin derivative
		526	MS^2^[526]: **482** (−44), 438 (−88), 508 (−18), 494 (−32), 466 (−60), 406 (−120), 349 (−177), 275 (−251), 263 (−263), 249 (−277)	Unidentified
		851	MS^2^[851]: **833** (−18), 709 (−*O*–PGU), 817 (−34), 691 (−160), 587 (−bifuhalol, −2), 435 (−2PGU−166), 455 (−3PGU−18), 761 (−90), 601 (−2PGU), 297 (−554), 583 (−268)	Fucofuropentaphlorethol
15	11.7	637	MS^2^[637]: **619** (−18), 496 (−141), 601 (−36), 335 (−2PGU−54), 577 (−60), 436 (−201), 471 (−166), 525 (−112), 575 (−44−18), 593 (−44), 555 (−84, −2), 419 (−218), 247 (−390), 373 (−bifuhalol, −2), 385 (−2PGU)	Pentafuhalol
16	12.2	610	MS^2^[610]: **566** (−44), 592 (−18), 449 (−161), 534 (−76), 462 (−148), 367 (−243), 229 (−381), 245 (−365), 309 (−301), 496 (−114)	Unidentified
		317	MS^2^[317]: **299** (−18), 274 (−43), 245 (−72), 259 (−58), 194 (−123), 125 (−192)	Phlorotannin derivative
		711	MS^2^[711]: **631** (−80), 693 (−18), 565 (−146), 639 (−72), 675 (−36), 395 (−316), 313 (−398), 469 (−242), 427 (−284), 371 (−340), 267 (−444), 229 (−482), 479 (−232), 513 (−198)	Phlorotannin derivative
		899	MS^2^[899]: **881** (−18), 863 (−36), 741 (−158), 755 (−PGU−18), 693 (−206), 759 (−140), 471 (−428), 453 (−446), 371 (−528), 263 (−636), 507 (−3PGU−18), 565 (−334), 649 (−2PGU)	Phlorotannin derivative
17	12.5	527	MS^2^[527]: **509** (−18), 483 (−44), 465 (−44−18), 437 (−90), 385 (−*O*–PGU), 261 (−bifuhalol), 401 (−PGU), 341 (−186), 491 (−36), 421 (−106), 455 (−72), 279 (−2PGU), 247 (−280)	Phlorotannin derivative
		635	MS^2^[635]: **575** (−60), 617 (−18), 335 (−300), 557 (−78), 369 (−bifuhalol), 509 (−PGU), 493 (−*O*–PGU), 457 (−178), 473 (−162), 273 (−2PGU−114), 229 (−406)	Phlorotannin derivative
		719	MS^2^[719]: **701** (−60), 553 (−166), 478 (−241), 460 (−259), 496 (−223), 683 (−36), 319 (−400), 331 (−388), 371 (−348), 249 (−3PGU−96)	Phlorotannin derivative
18	13.0	723	MS^2^[723]: **677** (−60), 695 (−28), 705 (−18), 659 (−64), 583 (−140), 356 (−367), 339 (−384), 477 (−246)	Unidentified
		587	MS^2^[587]: **507** (−80)	Unidentified
19	13.7	837	MS^2^[837]: **789** (−48), 747 (−90), 619 (−218), 581 (−256), 453 (−384), 265 (−572)	Unidentified
20	14.4	950	MS^2^[950]: **904** (−46), 696 (−254)	Unidentified
		667	MS^2^[667]: **649** (−18), 635 (−32), 605 (−44−18), 379 (−288), 521 (−146), 507 (−160), 451 (−216), 317 (−350), 297 (−370), 271 (−396)	Unidentified
21	14.6	587	MS^2^[587]: **507** (−80)	Unidentified

* Fragments are arranged in descending order of relative abundance with bold values highlighting the most abundant fragment.

**Table 6:** Independent variables and their coded levels used in the BBD.

Symbols	Independent Variables	Levels		
		−1	0	1
*X*_1_	Solvent concentration (% v/v)	30	50	70
*X*_2_	Solvent-solid ratio (mL/g)	30	50	70
*X*_3_	Temperature (°C)	15	25	35

**Table 7:** Box-Behnken experimental design matrix and the experimental and predicted values observed for TPhC.

Extract No.	Independent Variables			TPhC (mg PGE/g DS)	
	*X*_1_	*X*_2_	*X*_3_	Experimental	Predicted
1	30	30	25	2.21	2.19
2	30	70	25	2.13	2.12
3	70	30	25	2.55	2.56
4	70	70	25	2.94	2.97
5	50	30	15	2.56	2.54
6	50	30	35	2.53	2.57
7	50	70	15	2.73	2.69
8	50	70	35	2.72	2.74
9	30	50	15	1.98	2.03
10	70	50	15	2.67	2.68
11	30	50	35	2.12	2.11
12	70	50	35	2.73	2.68
13	50	50	25	2.63	2.69
14	50	50	25	2.70	2.69
15	50	50	25	2.73	2.69
